# Noninvasive prenatal diagnosis of monogenic disorders based on direct haplotype phasing through targeted linked-read sequencing

**DOI:** 10.1186/s12920-021-01091-x

**Published:** 2021-10-09

**Authors:** Chao Chen, Min Chen, Yaping Zhu, Lu Jiang, Jia Li, Yaoshen Wang, Zhe Lu, Fengyu Guo, Hairong Wang, Zhiyu Peng, Yun Yang, Jun Sun

**Affiliations:** 1 BGI Genomics, BGI-Shenzhen, Shenzhen, 518083 China; 2 Tianjin Medical Laboratory, BGI-Tianjin, BGI-Shenzhen, Tianjin, 300308 China; 3 Department of Fetal Medicine and Prenatal Diagnosis, The Third Affiliated Hospital of Guangzhou Medical University, Guangzhou, 510150 China; 4 BGI-Wuhan Clinical Laboratories, BGI-Shenzhen, Wuhan, 430074 China; 5 Department of Obstetrics and Gynecology, The Second Affiliated Hospital of Zhengzhou University, Zhengzhou, 450052 China

**Keywords:** Noninvasive prenatal diagnosis, Direct haplotype phasing, Targeted linked-read sequencing, Monogenic disease

## Abstract

**Background:**

Though massively parallel sequencing has been widely applied to noninvasive prenatal screen for common trisomy, the clinical use of massively parallel sequencing to noninvasive prenatal diagnose monogenic disorders is limited. This study was to develop a method for directly determining paternal haplotypes for noninvasive prenatal diagnosis of monogenic disorders without requiring proband’s samples.

**Methods:**

The study recruited 40 families at high risk for autosomal recessive diseases. The targeted linked-read sequencing was performed on high molecular weight (HMW) DNA of parents using customized probes designed to capture targeted genes and single-nucleotide polymorphisms (SNPs) distributed within 1Mb flanking region of targeted genes. Plasma DNA from pregnant mothers also underwent targeted sequencing using the same probes to determine fetal haplotypes according to parental haplotypes. The results were further confirmed by invasive prenatal diagnosis.

**Results:**

Seventy-eight parental haplotypes of targeted gene were successfully determined by targeted linked-read sequencing. The predicted fetal inheritance of variant was correctly deduced in 38 families in which the variants had been confirmed by invasive prenatal diagnosis. Two families were determined to be no-call.

**Conclusions:**

Targeted linked-read sequencing method demonstrated to be an effective means to phase personal haplotype for noninvasive prenatal diagnosis of monogenic disorders.

**Supplementary Information:**

The online version contains supplementary material available at 10.1186/s12920-021-01091-x.

## Background

The discovery of cell-free fetal circulating DNA (cff-DNA) in maternal blood and the rapid advances of massively parallel sequencing (MPS) have provided an unprecedented opportunity to perform the prenatal genetic testing of common fetal aneuploidies and single-gene diseases. Though MPS has been widely applied to screen for fetal trisomy 21, 18 and 13 [1], the clinical use of MPS to diagnose monogenic disorders is limited [2]. Several studies have been conducted to develop noninvasive prenatal diagnosis (NIPD) for monogenic disease using various technologies such as real-time polymerase chain reaction (PCR), amplification at lower denaturation temperature-PCR, digital PCR, circulating single-molecule amplification and resequencing technology [3, 4] and MPS. These studies were confined to exclude paternally inherited [5] and detect de novo variants[6] based on variant-specific assays due to the strong interference of maternal background signal. The relative haplotype dosage approach has been demonstrated to detect parental inherited variants at the same time. Our group has employed a proband-based method for resolving parental haplotypes and successfully applied this method to NIPD of Duchenne muscular dystrophy (DMD) [7], congenital adrenal hyperplasia (CAH) [8], maple syrup urine disease (MSUD) [9], hyperphenylalaninemia [10] and spinal muscular atrophy (SMA) [11]. This phasing information makes it possible to measure the haplotype dosage imbalance in maternal plasma DNA. The advantage of relative haplotype dosage approach is that analysis is independent of variant types. While, the method needs proband’s samples to phase parental haplotypes, which hampers the application of NIPD to monogenic diseases in clinical practice. The haplotype phasing is a critical step for haplotype-based NIPD of monogenic disorders. Serval studies have reported specific haplotype building methods such as clone pool dilution sequencing [12], contiguity-preserving transposition sequencing [13], targeted locus amplification (TLA) [14], HaploSeq [15] and long fragment read (LFR) technology [16]. These approaches need complex experimental operations and are time consuming and associated with a low success rate. These limitations can be problematic for identifying single gene disorders. Population data-based personal haplotype phasing overcomes the above drawbacks. The population-based method is based on reference population with genotyping data of unrelated individuals and the accuracy of NIPD is only 80%, which is lower than the experimental methods [17]. In order to further improve the success rate and accuracy of haplotype phasing, microfluidics-based linked-read sequencing technology and TLA-based phasing were utilized to phase parental DNA directly [18, 19]. The former approach combined the whole-genome sequencing (WGS) and linked-read sequencing method and succeeded in predicting fetal inherited variants in 12 of 13 pregnancies. The informative sequencing depth (40x) of WGS and the expensive experimental reagents restricted its clinical practice for NIPD [18]. Targeted TLA-based phasing approach is also subject to the complex acquisition of TLA template and customized target kit for NIPD which is inconvenient. A customized probe which covers dozens of common single gene disorders in China is used for haplotype-based NIPD. Therefore, we speculated that the linked-read sequencing combined with targeted sequencing using the above probes would expand the list of single gene disorders and reduce the cost compared with the whole-genome sequencing.

In our previous study, we demonstrated direct haplotyping of NIPD based on linked‐read sequencing is accurate for the prediction of fetal pathogenic variants of DMD [20]. The objectives of this study are to investigate the feasibility and accuracy of targeted linked-read sequencing in six different types of autosomal recessive diseases. We analyzed 40 families at high risk for six kinds of autosomal recessive diseases and showed that direct haplotype phasing of parental high molecular weight (HMW) DNA is feasible using targeted linked-read sequencing of target genes. Targeted sequencing of maternal plasma DNA combined with the parental haplotype information were interpreted to determine the inherited variants in fetus. Our approach might be a cost-effective and applicable method for NIPD of autosomal recessive monogenic disorders in clinical settings.

## Methods

### Sample collection

We recruited 40 families at high risk for autosomal recessive diseases, including 13 methylmalonic acidemia (MMA) families, 12 β-thalassemia families, 8 phenylketonuria (PKU) families, 5 α-thalassemia families, 1 autosomal recessive polycystic kidney disease (ARPKD) family and 1 autosomal recessive deafness-1A (DFNB1A) family caused by pathogenic variants of *GJB2* gene. The variants have been identified in all families (Table 1). All participants provided written informed consent to join in the study. The ethics committee of the participating hospitals and the Institutional Review Board of BGI approved the conduct of this study (BGI-IRB No 17080-T1).

**Table 1:** Clinical information of the participating families

Family	Disease	Gene	Genotypes of the Trios			GA	FF (%)
			Mat	Pat	Fetus (Mat/Pat)		
F01	β-thalassemia	*HBB*	c.316-197C>T /N	c.-78A>G/N	N/N	12^+4^	9.3
F02	β-thalassemia	*HBB*	c.126_129delCTTT/N	c.126_129delCTTT/N	N/N	20^+5^	15.9
F03	β-thalassemia	*HBB*	c.126_129delCTTT/N	c.-78A>G/N	c.126_129delCTTT/c.-78A>G	12^+3^	15.4
F04	β-thalassemia	*HBB*	c.316-197C>T /N	c.126_129delCTTT/N	N/c.126_129delCTTT	18	12.1
F05	β-thalassemia	*HBB*	c.126_129delCTTT/N	c.316-197C>T /N	c.126_129delCTTT/N	13^+6^	20.6
F06	β-thalassemia	*HBB*	c.216_217insA/T/N	c.126_129delCTTT/N	c.216_217insA/T/ c.126_129delCTTT	13^+2^	26.8
F07	β-thalassemia	*HBB*	c.79G>A/N	c.126_129delCTTT/N	N/c.126_129delCTTT	11^+3^	12.3
F08	β-thalassemia	*HBB*	c.126_129delCTTT/N	c.316-197C>T/N	c.126_129delCTTT/N	12^+3^	16.5
F09	β-thalassemia	*HBB*	c.52A>T/N	c.84_85insC/N	c.52A>T/N	12^+1^	27.7
F10	β-thalassemia	*HBB*	c.126_129delCTTT/N	c.79G>A/N	c.126_129delCTTT/c.79G>A	11^+1^	17.7
F11	β-thalassemia	*HBB*	c.126_129delCTTT/N	c.126_129delCTTT/N	c.126_129delCTTT/c.126_129delCTTT	17	8.1
F12	β-thalassemia	*HBB*	c.126_129delCTTT/N	c.126_129delCTTT/N	N/c.126_129delCTTT	17	9.7
F13	α-thalassemia	*HBA*	- -^SEA^/N	- -^SEA^/N	- -^SEA^/- -^SEA^	13^+3^	15.7
F14	α-thalassemia	*HBA*	- -^SEA^/N	- -^SEA^/N	N/N	11^+6^	13.7
F15	α-thalassemia	*HBA*	- -^SEA^/N	- -^SEA^/N	N/- -^SEA^	12^+4^	17.5
F16	α-thalassemia	*HBA*	- -^SEA^/N	- -^SEA^/N	- -^SEA^/- -^SEA^	11^+3^	23.5
F17	α-thalassemia	*HBA*	- -^SEA^/N	c.369C>G/N	- -^SEA^/c.369C>G	18	6.7
F18	MMA	*MMACHC*	c.609G>A/N	c.609G>A/N	c.609G>A/N	19	16.5
F19	MMA	*MMACHC*	c.656-658delAGA/N	c.609G>A/N	N/c.609G>A	18	14.2
F20	MMA	*MMACHC*	c.609G>A/N	c.656-658delAGA/N	N/N	16	12.8
F21	MMA	*MMACHC*	c.656-658delAGA/N	c.609G>A/N	N/N	17	10.4
F22	MMA	*MMACHC*	c.80A>G/N	c.609G>A/N	c.80A>G/N	17	10.2
F23	MMA	*MMACHC*	c.609G>A/N	c.441TG[2]/N	c.609G>A/c.441TG[2]	17	10.1
F24	MMA	*MMACHC*	c.609G>A/N	c.609G>A/N	N/N	18	17.8
F25	MMA	*MMACHC*	c.80A>G/N	c.609G>A/N	N/N	17	13.7
F26	MMA	*MMACHC*	c.609G>A/N	c.658-660delAAG/N	c.609G>A/c.658-660delAAG	17	9.8
F27	MMA	*MMACHC*	c.609G>A/N	c.445-446delTG/N	N/N	17	10.4
F28	MMA	*MMACHC*	c.482G>A/N	c.445-446delTG/N	N/N	17	8.2
F29	MMA	*MMACHC*	c.315C>G/N	c.609G>A/N	c.315C>G/N	16	6.5
F30	MMA	*MMACHC*	c.609G>A/N	c.609G>A/N	N/N	17^+5^	8.0
F31	PKU	*PAH*	c.1197A>T/N	c.764T>C/N	c.1197A>T/c.764T>C	18	7.3
F32	PKU	*PAH*	c.992T>C/N	c.770G>T/N	N/c.770G>T	17	7.5
F33	PKU	*PAH*	c.1045T>G/N	c.728G>A/N	N/N	18	11.3
F34	PKU	*PAH*	c.728G>A/N	c.611A>G/N	N/N	20	5.9
F35	PKU	*PAH*	c.977G>A/N	c.1238G>C/N	c.977G>A/N	17	21.2
F36	PKU	*PAH*	c.473G>A/N	c.208_210delTCT	c.473G>A/c.208_210delTCT	18	12.8
F37	PKU	*PAH*	c.1223G>A/N	c.727C>T/N	N/N	12	8.5
F38	PKU	*PAH*	c.728G>A/N	c.721C>T/N	c.728G>A/c.721C>T	12	7.2
F39	ARPKD	*PKHD1*	c.11042T>G/N	c.5137G>T /N	N/c.5137G>T	12^+6^	15.0
F40	DFNB1A	*GJB2*	c.235delC/N	c.299-300delAT/N	c.235delC/N	13^+1^	15.3

*FF* fetal fraction, *GA* gestational age, *N* Normal, *PKU* phenylketonuria, *MMA* methylmalonic academia, *ARPKD* autosomal recessive polycystic kidney disease, *DFNB1A* autosomal recessive deafness-1A

### Target capture probe design

The targeted enrichment of DNA libraries was performed according to the custom-designed SeqCap EZ Choice Library (NimbleGen, Roche) protocol. The capture probes (NimbleGen, Roche) targeting the whole genes of *HBB*, *HBA1*, *HBA2*, and highly heterozygous SNPs within 1Mb flanking region of target genes were designed for NIPD of β-thalassemia and α-thalassemia. Another set of target capture probe was designed to cover the coding region and SNPs within 1Mb upstream and downstream regions of the interested genes, including *MMACHC* (MMA), *PAH* (PKU), *PKHD1* (ARPKD) and *GJB2* (DFNB1A).

### Targeted linked-read sequencing

HMW genomic DNA (gDNA) was extracted from stored blood using the Mag Attract HMW Kit (Qiagen, Germany). The size of HMW gDNA should be more than 50kb according to the pulse electrophoresis results. Then gDNA was processed with Chromium™ Genome v2 libraries (10x Genomics, USA). Long gDNA strands were partitioned in barcoded gel beads through a microfluidic device. Barcoded oligonucleotides in a gel bead bind randomly onto the long molecules and generate short fragments with the same barcode. The chance that two molecules were covering the same genomic locus on each gel bead is low, and the short fragments with the same barcode were considered to come from the same long molecule. Libraries of the barcoded fragments were prepared and captured using the customized probe. The prepared DNA library was then sequenced using an Illumina HiSeq2500 sequencer with a paired-end format of 101 bp or 150 bp.

### Variant calling and direct haplotype phasing

The barcoded libraries read were then processed with the Long Ranger pipeline (v.2.2.2) provided by 10x Genomics [21]. Reads associated with valid barcodes were aligned against the human genome 19 (Hg19) by using the Burrows-Wheeler Aligner (BWA) software [22]. Output files annotated with barcode and phasing information were generated and served as the reference haplotypes of the family for downstream analysis. The maternal plasma DNA sequencing reads were aligned against the reference hg19 using BWA. After duplicated reads were marked by the Picard Mark Duplicates tool, the GATK tools were applied to perform local realignment and base quality score recalibration [23].

The free Long Ranger (v.2.2.2) software was utilized to determine the parental haplotype in the interested region. Barcode information provides the clue to associate short reads to the original long input molecules. Variant-linked haplotype referred to those reads whose barcodes were consistent with the ones with variant alleles. In contrast, wild-linked haplotype denoted the reads carrying same barcode with the ones with wild-type alleles. The different haplotype blocks were linked with identified SNPs using the overlapping region. SNPs associated with the same haplotypes carrying the wild-type and variant alleles were used for the maternal plasma DNA analysis.

### The estimation of fetal fraction and NIPD of monogenic disorders

The evaluation of fetal fraction could be conducted according to the procedure reported before [8]. The haplotype related to variant and wild alleles was constructed based on targeted linked-read sequencing. The informative SNPs that were heterozygous in the mother but homozygous in the father were analyzed for maternal inheritance. On the contrary, the paternal inheritance analysis followed the opposite strategy with maternal inheritance analysis. We used hidden Markov model (HMM) to predict the most likely inherited haplotype using our previously reported algorithm [24]. The probabilities that the fetus inherited the pathogenic and non-pathogenic alleles were evaluated using the number of reads in maternal plasma and then considered as the HMM emission probabilities. The genetic map from the National Center for Biotechnology Information provided the genetic position of the SNPs in centimorgan (cM) and recombination rates between SNPs, these probabilities were regarded as HMM transition probabilities. Lastly, the Viterbi algorithm was utilized to predict the inherited haplotype in the fetus.

### Validation of NIPD

The samples obtained through invasive procedures including chorionic villus sampling (CVS) and amniocentesis were used for prenatal genetic diagnosis. After DNA extraction, Sanger sequencing, gap-PCR and reverse dot blot PCR for target variations were performed in a blind manner to further validate the accuracy of NIPD.

## Results

### Clinical information of the monogenic families

40 families at high risk for autosomal recessive diseases, including 13 MMA families, 12 β-thalassemia families, 8 PKU families, 5 α-thalassemia families, 1 ARPKD family and 1 DFNB1A family caused by pathogenic variants of *GJB2* gene were recruited. The gestational age (GA) of 40 pregnant women varied from 11^+1^ to 20^+5^ weeks, with a median GA of 15.5 weeks. The clinical information, variant loci and variant status of the 40 families are presented in Table 1.

### Targeted linked-reads sequencing

Targeted sequencing on the interested gene region was performed in plasma DNA samples from 40 pregnant women at different gestational weeks. The fetal fraction varied from 5.9 to 27.7%, with a mean fetal fraction of 13.2%, showing significant differences between individuals (Table 1). The targeted sequencing of gDNA samples showed the coverage was relatively consistent in the targeted genes, with a mean read depth of 402× (Additional file 1: Table S1). After data pre-processing and alignment, over 98% of the linked-reads were aligned to the hg19, an average of 50% of the bases were on-target (Additional file 1: Table S1). The summary statistics of alignment are presented in detail in Additional file 1: Table S1.

### Direct haplotype phasing

The 10x genomics barcoding technology allowed us to obtain long-range information by linking the short sequencing reads produced. There were two haplotypes, the pathogenic haplotype (P) and normal haplotype (N). The former referred to the reads whose alleles or barcodes were in consistence with variant-supporting reads at heterozygous SNP positions. While the latter represented those reads whose alleles were opposite to the variant-supporting reads at heterozygous SNP positions. The two haplotypes of were directly determined by linking the haplotype blocks assembled by the barcoded reads for all parental gDNA. N50 phase-block length represents the contiguity achieved in the experimental haplotyping, the average length of N50 phase-block was 1Mb (range 413.04 kb~3.54 Mb). N50 phase block, phase block across the target region and longest phase block for the 40 families is depicted in Table 2 and Additional file 1: Table S1. The number of SNPs in the phase blocks used for phasing ranged from 3 to 2418 SNPs, with a mean of 1006 (Table 2). All variants carried by family members were initially detected by the targeted linked-read sequencing and verified to be concordant with those from the MPS data. The paternal haplotypes phasing of F27 and F36 failed, because the haplotype block cannot cover the pathogenic variants. Therefore, the NIPD analysis is not required for failed phasing individuals (pF27 and pF36).

**Table 2:** Parental haplotypes phasing data

Family	Sample	Gene	Phase block across target region	Phasing block size(kb)	No. of SNPs across the block
F01	mat	*HBB*	chr11:4249489-6238960	1989.5	2367
	pat	*HBB*	chr11:4269280-5761797	1492.5	1469
F02	mat	*HBB*	chr11:4366798-6246383	1879.6	1803
	pat	*HBB*	chr11:4366798-6237565	1870.8	1655
F03	mat	*HBB*	chr11:4249238-5884595	1635.4	1716
	pat	*HBB*	chr11:4346064-6121271	1775.2	1972
F04	mat	*HBB*	chr11:4587676-6243982	1656.3	2308
	pat	*HBB*	chr11:4905140-6216304	1311.2	1644
F05	mat	*HBB*	chr11:5192535-5900085	707.6	955
	pat	*HBB*	chr11:4249095-5450493	1201.4	1359
F06	mat	*HBB*	chr11:4852009-5555972	704.0	741
	pat	*HBB*	chr11:5196669-6082903	886.2	1541
F07	mat	*HBB*	chr11:4697080-6239344	1542.3	1810
	pat	*HBB*	chr11:4306665-6246051	1939.4	2043
F08	mat	*HBB*	chr11:4936613-6116142	1179.5	1544
	pat	*HBB*	chr11:4249126-5771915	1522.8	1369
F09	mat	*HBB*	chr11:4436676-6239344	1802.7	1681
	pat	*HBB*	chr11:4249163-6090372	1841.2	2247
F10	mat	*HBB*	chr11:4249271-6237565	1988.3	1666
	pat	*HBB*	chr11:4249031-6037803	1788.8	1733
F11	mat	*HBB*	chr11:4345701-5647166	1301.5	1202
	pat	*HBB*	chr11:4389404-5719251	1329.8	1450
F12	mat	*HBB*	chr11:4249095-6239344	1990.2	2301
	pat	*HBB*	chr11:4387760-6121428	1733.7	2418
F13	mat	*HBA*	chr16:60185-679412	619.2	284
	pat	*HBA*	chr16:60185-1225628	1165.4	937
F14	mat	*HBA*	chr16:186950-1216997	1030.0	606
	pat	*HBA*	chr16:132246-612607	480.4	251
F15	mat	*HBA*	chr16:94080-1225184	1131.1	899
	pat	*HBA*	chr16:74039-1197612	1123.6	687
F16	mat	*HBA*	chr16:79811-1223722	1143.9	883
	pat	*HBA*	chr16:60185-460830	400.6	339
F17	mat	*HBA*	chr16:60185-1192620	1132.4	1045
	pat	*HBA*	chr16:60291-1225184	1164.9	1010
F18	mat	*MMACHC*	chr1: 44966837-46952164	1985.3	1599
	pat	*MMACHC*	chr1: 44972309-46972958	2000.6	926
F19	mat	*MMACHC*	chr1: 45513754-46973454	1459.7	440
	pat	*MMACHC*	chr1: 44979498-46975877	1996.3	831
F20	mat	*MMACHC*	chr1: 45767431-46206444	439.0	119
	pat	*MMACHC*	chr1: 45386861-46503217	1116.3	247
F21	mat	*MMACHC*	chr1: 45765523-46975294	1209.8	457
	pat	*MMACHC*	chr1:44967323-46975877	2008.6	812
F22	mat	*MMACHC*	chr1:45762749-46722939	960.2	445
	pat	*MMACHC*	chr1:45701916-46097939	396.0	161
F23	mat	*MMACHC*	chr1:45738336-46975450	1237.1	729
	pat	*MMACHC*	chr1:44967431-46975877	2008.4	1228
F24	mat	*MMACHC*	chr1:45947353-46095125	147.8	27
	pat	*MMACHC*	chr1:45775550-46605728	830.2	609
F25	mat	*MMACHC*	chr1:45765523-46053981	288.5	156
	pat	*MMACHC*	chr1:45765523-45982693	217.2	41
F26	mat	*MMACHC*	chr1:45767431-46975877	1208.4	691
	pat	*MMACHC*	chr1:45762749-46975877	1213.1	684
F27	mat	*MMACHC*	chr1:45683746-46645681	961.9	572
	pat	*MMACHC*	chr1:45962137-45974407	12.3	3
F28	mat	*MMACHC*	chr1:44967323-45974520	1007.2	595
	pat	*MMACHC*	chr1:44967323-46691245	1723.9	1149
F29	mat	*MMACHC*	chr1:45640368-46975877	1335.5	599
	pat	*MMACHC*	chr1:44973546-46975877	2002.3	1185
F30	mat	*MMACHC*	chr1:44967825-46975877	2008.1	1082
	pat	*MMACHC*	chr1:45683419-46924563	1241.1	685
F31	mat	*PAH*	chr12:103214192-104013534	799.3	301
	pat	*PAH*	chr12:102252463-104225303	1972.8	1299
F32	mat	*PAH*	chr12:102241500-104309559	2068.1	1300
	pat	*PAH*	chr12:102240964-104261374	2020.4	1094
F33	mat	*PAH*	chr12:102240964-103276441	1035.5	555
	pat	*PAH*	chr12:102241500-104173880	1932.4	1048
F34	mat	*PAH*	chr12:102618568-104309712	1691.1	1095
	pat	*PAH*	chr12:102728895-104272113	1543.2	696
F35	mat	*PAH*	chr12:102894838-103267467	372.6	136
	pat	*PAH*	chr12:103075411-104309383	1234.0	1069
F36	mat	*PAH*	chr12:102248565-104275721	2027.2	1189
	pat	*PAH*	chr12:103105959-103274915	169.0	106
F37	mat	*PAH*	chr12:102321986-103791220	1469.2	984
	pat	*PAH*	chr12:102710699-104300441	1589.7	1062
F38	mat	*PAH*	chr12:102240964-103623855	1382.9	619
	pat	*PAH*	chr12:102240964-104304705	2063.7	1246
F39	mat	*PKHD1*	chr6:50968947-52950047	1981.1	1347
	pat	*PKHD1*	chr6:50982112-52905592	1923.5	985
F40	mat	*GJB2*	chr13:20687773-20802900	115.1	93
	pat	*GJB2*	chr13:20676993-21122165	445.2	279

### Noninvasive prenatal diagnosis

**Fig. 1.**
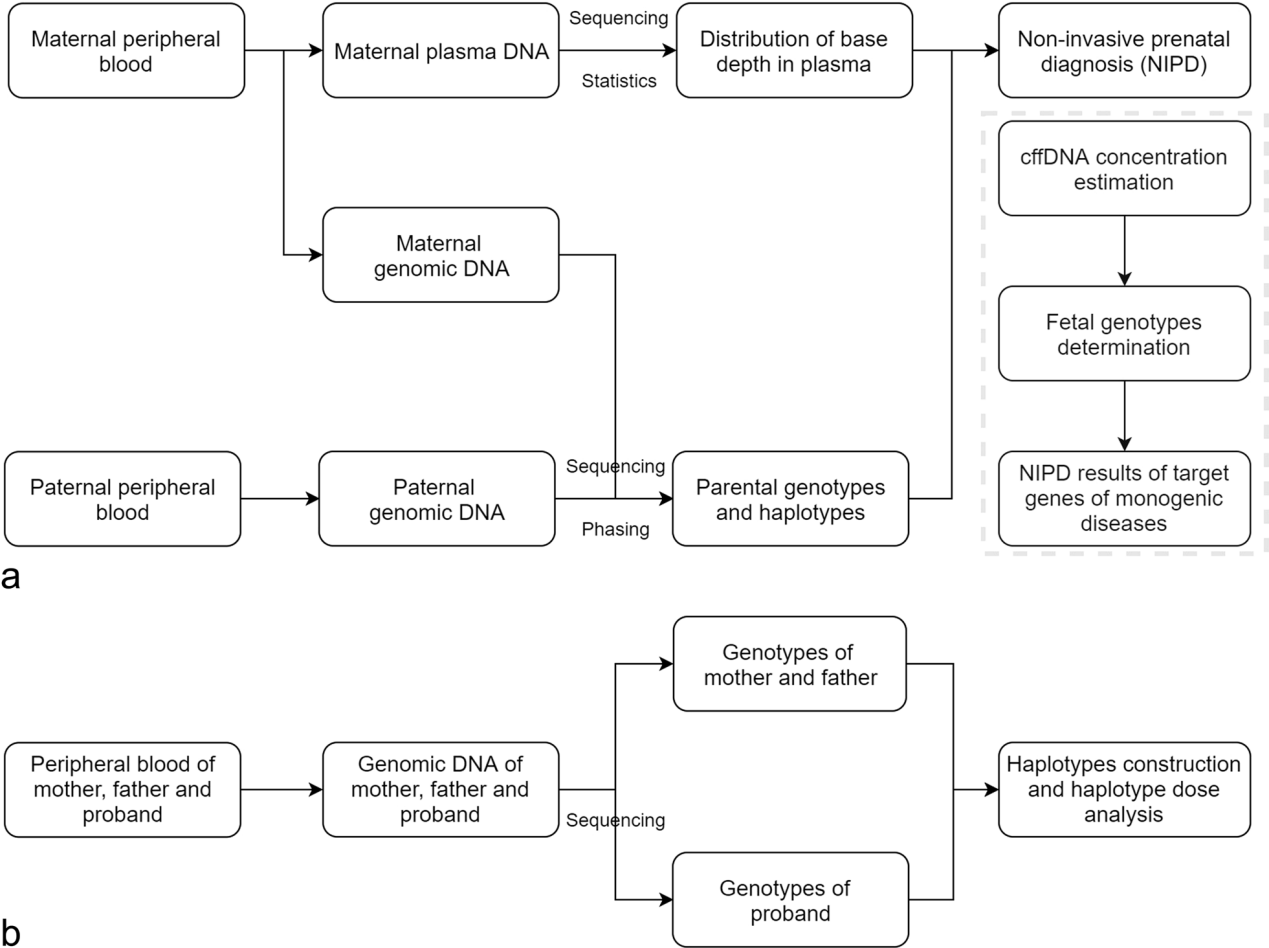
The flow charts of targeted linked-read sequencing and proband-based indirect phasing. **a** Parental genotypes and haplotype determination, prediction of fetal haplotype and noninvasive prenatal diagnosis of monogenic diseases using the targeted linked-read sequencing method. **b** Parental and proband's genotype and haplotype determination, prediction of fetal haplotype and noninvasive prenatal diagnosis of monogenic diseases using the proband-based indirect phasing method

As shown in the NIPD flowchart (Figure 1), maternal and paternal haplotypes were first established using target-region sequencing data and the HMM and Viterbi algorithm was then applied to predict fetal haplotypes. Our goal was to precisely infer the fetal genotypes at pathogenic sites, not to correctly infer the haplotypes of all SNP markers flanking the target gene. Therefore, the specific rules [25] were set to determine the fetal genotype at the pathogenic site after obtaining the optimal path of the fetal haplotype block via the Viterbi algorithm. If the path contains only one halotype block (pathogenic or normal) and the block spans the target gene, the fetal genotype at the pathogenic site is the state of the haplotype block that spans the target gene. If the path contains two haplotype blocks (pathogenic and normal) and only one haplotype block spans the target gene, the fetal genotype at the pathogenic site is the state of the haplotype block that spans the target gene (for example, mF04 and mF06). If two haplotype block (pathogenic and normal) exists inside the target gene, the fetal genotype at the pathogenic site is determined as no-call (for example, mF36). A confidence score (CS) [25] was introduced into our algorithm to quantify the probability of obtaining the correct results for NIPD. The CS was calculated using the fetal fraction, sequencing depth of maternal plasma and number of parental informative SNPs as inputs for computational simulation. The detailed method can be referred to the published literature [25]. The condition that the CS was less than 0.99 was defined as no-call.

The NIPD results exhibited that 38 fetuses had both alleles detected; of these 38 fetuses, 11 were affected, 15 were carriers and 12 were normal. (Table 3, Additional file 2: Figure S1, Additional file 3: Figure S2 and Additional file 4: Figure S3). For F27, only one normal haplotype inherited from mother can be inferred by NIPD. For F36, we cannot predict fetal haplotypes inherited from parents.

**Table 3:** The NIPD results

Family	Gene	No. of Maternal Informative SNPs	No. of Paternal Informative SNPs	CS_mat_ (%)	CS_pat_ (%)	NIPD (mat/pat)	Invasive prenatal diagnosis (mat/pat)
F01	*HBB*	1260	305	100	100	N/N	N/N
F02	*HBB*	1073	607	100	100	N/N	N/N
F03	*HBB*	521	566	100	100	c.126_129delCTTT/c.-78A>G	c.126_129delCTTT/c.-78A>G
F04	*HBB*	394	317	100	100	N/c.126_129delCTTT	N/c.126_129delCTTT
F05	*HBB*	255	555	100	100	c.126_129delCTTT/N	c.126_129delCTTT/N
F06	*HBB*	268	453	100	100	c.216_217insA/T/ c.126_129delCTTT	c.216_217insA/T/ c.126_129delCTTT
F07	*HBB*	697	695	100	100	N/c.126_129delCTTT	N/c.126_129delCTTT
F08	*HBB*	636	442	100	100	c.126_129delCTTT/N	c.126_129delCTTT/N
F09	*HBB*	669	553	100	100	c.52A>T/N	c.52A>T/N
F10	*HBB*	908	594	100	100	c.126_129delCTTT/c.79G>A	c.126_129delCTTT/c.79G>A
F11	*HBB*	603	380	100	100	c.126_129delCTTT/c.126_129delCTTT	c.126_129delCTTT/c.126_129delCTTT
F12	*HBB*	1029	550	100	100	N/c.126_129delCTTT	N/c.126_129delCTTT
F13	*HBA*	53	18	100	100	- -^SEA^/- -^SEA^	- -^SEA^/- -^SEA^
F14	*HBA*	235	52	100	100	N/N	N/N
F15	*HBA*	118	84	100	100	N/- -^SEA^	N/- -^SEA^
F16	*HBA*	193	78	100	100	- -^SEA^/- -^SEA^	- -^SEA^/- -^SEA^
F17	*HBA*	361	140	100	100	- -^SEA^/c.369C>G	- -^SEA^/c.369C>G
F18	*MMACHC*	775	228	100	100	c.609G>A/N	c.609G>A/N
F19	*MMACHC*	298	424	100	100	N/c.609G>A	N/c.609G>A
F20	*MMACHC*	97	175	100	100	N/N	N/N
F21	*MMACHC*	348	361	100	100	N/N	N/N
F22	*MMACHC*	285	49	100	100	c.80A>G/N	c.80A>G/N
F23	*MMACHC*	531	300	100	100	c.609G>A/c.441TG[2]	c.609G>A/c.441TG[2]
F24	*MMACHC*	15	420	100	100	N/N	N/N
F25	*MMACHC*	79	7	100	100	N/N	N/N
F26	*MMACHC*	492	107	100	100	c.609G>A/c.658-660delAAG	c.609G>A/c.658-660delAAG
F27	*MMACHC*	353	NA	100	NA	N/NA	N/N
F28	*MMACHC*	474	457	100	100	N/N	N/N
F29	*MMACHC*	319	469	100	100	c.315C>G/N	c.315C>G/N
F30	*MMACHC*	776	42	100	100	N/N	N/N
F31	*PAH*	69	321	100	100	c.1197A>T/c.764T>C	c.1197A>T/c.764T>C
F32	*PAH*	362	185	100	100	N/c.770G>T	N/c.770G>T
F33	*PAH*	161	147	100	100	N/N	N/N
F34	*PAH*	262	95	100	100	N/N	N/N
F35	*PAH*	13	174	100	100	c.977G>A/N	c.977G>A/N
F36	*PAH*	188	NA	100	NA	NC [*]/NA	c.473G>A/c.208_210delTCT
F37	*PAH*	261	164	100	100	N/N	N/N
F38	*PAH*	406	561	100	100	c.728G>A/c.721C>T	c.728G>A/c.721C>T
F39	*PKHD1*	971	267	100	100	N/c.5137G>T	N/c.5137G>T
F40	*GJB2*	29	53	100	100	c.235delC/N	c.235delC/N

*NC no-call, *NA* not applicable, *No.* number, *CS*_*mat*_ confidence score for fetal inheritance from maternal haplotype, *CS*_*pat*_ confidence score for fetal inheritance from paternal haplotype

The fetal genotypes inferred by NIPD were compared with direct sequencing results of fetal gDNA extracted from CVS or amniotic fluid cells to further validate the accuracy of NIPD. The results of NIPD were in concordant with invasive diagnosis and the standard genotype of captured sequencing (Table 3).

## Discussion

In our study, we applied the targeted linked-read sequencing method to resolve the parental haplotypes across a range of disease loci and successfully determined the fetal genotypes in 38 families, at risk for various single gene diseases. The previous method of NIPD needs the input of the genomics data of an affected family member and involves complex computational resources for indirectly phasing proband-based haplotype. As compared to the previous NIPD method, our targeted linked-read sequencing method may show certain advantages. Either genomics data from a proband or other family members may not be obligatory for deducing fetal variant status, or an additional capture probe. The new method may in particularly benefit the first pregnancy for those women carrying disease variants, due to lack of genomics information from other affected family members.

In recent years, several studies have utilized the direct haplotyping method to perform NIPD of single gene disorders [18, 23]. Hui et al conducted whole genome haplotyping method and resolved the parental haplotypes with the use of linked-read sequencing technology. They correctly deduced the fetal variant profiles in 12 out of 13 families at risk for a number of autosomal and X-linked diseases. However, the cost of whole genome haplotyping method is relatively high, which might limit its wide use in clinical settings. Vermeulen et al established the targeted locus amplification approach and phased heterozygous variants in selected genes, the method reduced the cost of whole genome haplotyping method and predicted fetal variant status with a high accuracy. Michael Parks utilized targeted capture enrichment of SNPs across a 6 Mb genomic window on chromosome 5 containing the *SMN1* gene and successfully deduced fetal variants by relative haplotype dosage with 100% accuracy [11]. However, customizing the targeted region might be a complex task, due to population frequency difference of SNPs across different ethnicities [26]. Our method is advantageous to the above-mentioned 2 direct phasing methods with respect to the cost-effectiveness and recombination prediction. The current NIPD practically requires maternal, paternal DNA and proband’s DNA samples, therefore, the cost of the current proband-dependent method is approximately $830. The major advantage of our method is that it bypassed the availability of the proband’s DNA which considerably reduced the cost to $700. Moreover, multiplexing of a barcoded library further reduces the cost of linked-read sequencing. The turnaround time of linked-read sequencing is 3 weeks, that is more time-consuming than that of the proband-based method but is still affordable for noninvasive prenatal diagnosis. One potential application of our method is NIPD of cystic fibrosis variants which are more relevant to other ethnicity. As demonstrated in this study, the capture probes should cover the whole CF transmembrane regulator (*CFTR*) gene and highly heterozygous SNPs within 1Mb flanking region of *CFTR*. With reduced cost, the targeted linked-read sequencing method is capable of NIPD of a wide range of monogenic disorders independently of proband sample.

Despite the advantages as mentioned above, our method still has certain limitations. First, the average percentage of bases on target is approximately 50%, the low on-target rate is a potential limitation of this linked-read target sequencing and may increase the sequencing cost. However, as compared to two other studies, in which the authors reported mean on-target rates of 30.7% and 32% [7, 19], our linked-read target sequencing outperformed the previously published methods. Second, the design of target region and capture probe is critical to successfully conduct targeted linked-read sequencing. There is no existent recommended guideline on the design of capture probes. Additionally, it’s essential to evaluate recombination hot spots surrounding the target region and include the results in the recombination adjustment [27]. Given the clinical applicability of linked-read sequencing hasn’t fully characterized, more researches are required to validate the readiness and effectiveness of this technique in the future.

## Conclusions

In summary, we have provided solid evidence that targeted linked-read sequencing method could be applied to the noninvasive assessment of a variety of fetal single gene diseases. The method is a cost-effective and could be widely adopted in clinical practice.

## Supplementary Information

**Additional file 1: Table S1**. Summary statistics of alignment

**Additional file 2: Figure S1**. The NIPD results of α-thalassemia and β-thalassemia

**Additional file 3: Figure S2**. The NIPD results of MMA

**Additional file 4: Figure S3**. The NIPD results of PKU, ARPKD and DFNB1A

## Data Availability

The datasets supporting the conclusions of this study are available from the corresponding author upon reasonable request. The raw datasets generated during the current study are not publicly available because it is possible that individual privacy could be compromised. The Human reference genome (Hg19) for alignment were obtained from UCSC (https://hgdownload.soe.ucsc.edu/goldenPath/hg19/bigZips/).

## Abbreviations

SNPs: Single-nucleotide polymorphisms
cff-DNA: Cell-free fetal circulating DNA
MPS: Massively parallel sequencing
NIPD: Noninvasive prenatal diagnosis
PCR: Polymerase chain reaction
DMD: Duchenne muscular dystrophy
CAH: Congenital adrenal hyperplasia
MSUD: Maple syrup urine disease
SMA: Spinal muscular atrophy
TLA: Targeted locus amplification
LFR: Long fragment read
WGS: Whole-genome sequencing
HMW: High molecular weight
MMA: Methylmalonic acidemia
PKU: Phenylketonuria
ARPKD: Autosomal recessive polycystic kidney disease
DFNB1A: Autosomal recessive deafness-1A
gDNA: Genomic DNA
BWA: Burrows–Wheeler Aligner
HMM: Hidden Markov model
cM: Centimorgan
CVS: Chorionic villus sampling
GA: Gestational age
CS: Confidence score
