# Supplementary material for: Noninvasive prenatal diagnosis of monogenic disorders based on direct haplotype phasing through targeted linked-read sequencing
Source: BMC Med Genomics. 2021 Oct 9;14:244. doi: 10.1186/s12920-021-01091-x (PMC8502361; doi:10.1186/s12920-021-01091-x)
Supplement: Supplementary file 1 — Additional file 1: Table S1. Summary statistics of alignment [file 12920_2021_1091_MOESM1_ESM.docx]

**Table S1. Summary statistics of alignment.**

| **Family** | **Sample** | **Germline mutations detected** | **SNPs phased** | **Longest phase block (bp)** | **Mean depth (x)** | **Mapped reads** | **On target bases** |
| --- | --- | --- | --- | --- | --- | --- | --- |
| F01 | mat | 407221 | 0.98 | 2457818 | 445.68 | 0.99 | 0.59 |
|  | pat | 443870 | 0.98 | 1964895 | 397.72 | 0.99 | 0.57 |
| F02 | mat | 713358 | 0.97 | 4791904 | 389.68 | 0.99 | 0.63 |
|  | pat | 333999 | 0.98 | 3285690 | 301.90 | 0.99 | 0.55 |
| F03 | mat | 450950 | 0.98 | 2330561 | 267.65 | 0.97 | 0.44 |
|  | pat | 390331 | 0.98 | 1916680 | 239.02 | 0.97 | 0.42 |
| F04 | mat | 432403 | 0.99 | 3388723 | 358.24 | 0.99 | 0.50 |
|  | pat | 414648 | 0.98 | 3146462 | 358.51 | 0.98 | 0.49 |
| F05 | mat | 490381 | 0.99 | 4886498 | 371.73 | 0.98 | 0.49 |
|  | pat | 460347 | 0.98 | 1994611 | 394.00 | 0.99 | 0.52 |
| F06 | mat | 580101 | 0.98 | 1700806 | 318.41 | 0.98 | 0.48 |
|  | pat | 554116 | 0.98 | 1555863 | 290.42 | 0.98 | 0.48 |
| F07 | mat | 408038 | 0.99 | 3187453 | 480.14 | 0.99 | 0.53 |
|  | pat | 444835 | 0.98 | 3699899 | 474.81 | 0.99 | 0.54 |
| F08 | mat | 381936 | 0.98 | 1990271 | 232.12 | 0.97 | 0.48 |
|  | pat | 430631 | 0.98 | 1545527 | 255.03 | 0.97 | 0.48 |
| F09 | mat | 438953 | 0.98 | 2455406 | 313.22 | 0.97 | 0.49 |
|  | pat | 338872 | 0.98 | 1821283 | 128.47 | 0.96 | 0.45 |
| F10 | mat | 461300 | 0.98 | 3392119 | 461.68 | 0.99 | 0.54 |
|  | pat | 438675 | 0.98 | 2950017 | 466.99 | 0.99 | 0.53 |
| F11 | mat | 459246 | 0.98 | 2975148 | 316.35 | 0.98 | 0.46 |
|  | pat | 470989 | 0.98 | 2425038 | 262.04 | 0.97 | 0.43 |
| F12 | mat | 453039 | 0.99 | 3872750 | 284.68 | 0.97 | 0.44 |
|  | pat | 436031 | 0.98 | 2409437 | 175.01 | 0.96 | 0.41 |
| F13 | mat | 638675 | 0.98 | 1615372 | 490.19 | 0.99 | 0.57 |
|  | pat | 539280 | 0.98 | 1449894 | 352.68 | 0.99 | 0.58 |
| F14 | mat | 544819 | 0.98 | 1825526 | 362.53 | 0.99 | 0.58 |
|  | pat | 437990 | 0.97 | 1582377 | 342.17 | 0.99 | 0.60 |
| F15 | mat | 297277 | 0.98 | 2477620 | 330.22 | 0.98 | 0.44 |
|  | pat | 303364 | 0.98 | 2047551 | 305.85 | 0.98 | 0.44 |
| F16 | mat | 359650 | 0.98 | 2430088 | 364.28 | 0.99 | 0.50 |
|  | pat | 380955 | 0.98 | 2326635 | 339.82 | 0.99 | 0.49 |
| F17 | mat | 351175 | 0.99 | 3493638 | 214.56 | 0.96 | 0.28 |
|  | pat | 339473 | 0.98 | 2505987 | 151.11 | 0.96 | 0.27 |
| F18 | mat | 287840 | 1.00 | 6761228 | 4568.15 | 0.94 | 0.36 |
|  | pat | 90272 | 0.96 | 2477620 | 45.21 | 0.97 | 0.37 |
| F19 | mat | 303519 | 0.99 | 4346114 | 1077.71 | 0.99 | 0.41 |
|  | pat | 395527 | 0.99 | 4901983 | 1051.07 | 0.99 | 0.41 |
| F20 | mat | 87079 | 0.95 | 4098672 | 48.04 | 0.96 | 0.40 |
|  | pat | 97495 | 0.96 | 2385044 | 48.94 | 0.97 | 0.40 |
| F21 | mat | 428027 | 0.98 | 2433770 | 330.12 | 0.99 | 0.55 |
|  | pat | 353942 | 0.97 | 3030490 | 328.40 | 0.99 | 0.55 |
| F22 | mat | 391655 | 0.98 | 2856525 | 346.58 | 0.99 | 0.56 |
|  | pat | 415588 | 0.98 | 2212936 | 384.40 | 0.99 | 0.56 |
| F23 | mat | 518230 | 0.98 | 1881338 | 549.26 | 0.99 | 0.57 |
|  | pat | 417337 | 0.98 | 2060787 | 451.52 | 0.99 | 0.56 |
| F24 | mat | 449093 | 0.98 | 2163747 | 403.92 | 0.99 | 0.56 |
|  | pat | 454991 | 0.98 | 1494345 | 432.86 | 0.99 | 0.55 |
| F25 | mat | 420699 | 0.98 | 2005063 | 490.97 | 0.98 | 0.57 |
|  | pat | 420115 | 0.98 | 1987695 | 438.45 | 0.99 | 0.55 |
| F26 | mat | 418934 | 0.98 | 2024087 | 417.34 | 0.97 | 0.55 |
|  | pat | 379253 | 0.98 | 2422978 | 429.15 | 0.97 | 0.55 |
| F27 | mat | 393700 | 0.98 | 2482506 | 458.14 | 0.98 | 0.55 |
|  | pat | 365983 | 0.98 | 2011286 | 372.19 | 0.97 | 0.55 |
| F28 | mat | 504620 | 0.98 | 3243904 | 547.71 | 0.99 | 0.55 |
|  | pat | 477269 | 0.98 | 3026726 | 527.63 | 0.99 | 0.56 |
| F29 | mat | 367034 | 0.98 | 4024699 | 364.87 | 0.99 | 0.56 |
|  | pat | 374917 | 0.98 | 3359765 | 395.28 | 0.99 | 0.56 |
| F30 | mat | 378162 | 0.99 | 4897124 | 466.76 | 0.99 | 0.56 |
|  | pat | 428023 | 0.98 | 2245625 | 451.36 | 0.99 | 0.55 |
| F31 | mat | 320227 | 0.98 | 2701264 | 218.75 | 0.98 | 0.48 |
|  | pat | 351260 | 0.98 | 2967627 | 308.05 | 0.98 | 0.52 |
| F32 | mat | 320234 | 0.98 | 4634780 | 294.69 | 0.98 | 0.52 |
|  | pat | 362156 | 0.98 | 2769999 | 266.93 | 0.98 | 0.49 |
| F33 | mat | 461140 | 0.98 | 1774495 | 303.16 | 0.99 | 0.53 |
|  | pat | 363825 | 0.98 | 2588003 | 320.77 | 0.99 | 0.53 |
| F34 | mat | 365983 | 0.98 | 2024087 | 318.29 | 0.98 | 0.51 |
|  | pat | 459331 | 0.98 | 3232678 | 330.26 | 0.97 | 0.49 |
| F35 | mat | 428643 | 0.98 | 2851311 | 268.00 | 0.97 | 0.48 |
|  | pat | 317392 | 0.98 | 1875599 | 246.94 | 0.97 | 0.48 |
| F36 | mat | 453091 | 0.98 | 3326125 | 338.50 | 0.98 | 0.54 |
|  | pat | 619599 | 0.98 | 2181933 | 302.73 | 0.98 | 0.55 |
| F37 | mat | 468909 | 0.98 | 3032176 | 320.02 | 0.98 | 0.53 |
|  | pat | 489369 | 0.98 | 1881728 | 318.93 | 0.98 | 0.52 |
| F38 | mat | 542316 | 0.98 | 2203804 | 395.23 | 0.99 | 0.54 |
|  | pat | 443543 | 0.98 | 2464306 | 327.21 | 0.98 | 0.51 |
| F39 | mat | 274995 | 0.98 | 3339050 | 161.54 | 0.97 | 0.51 |
|  | pat | 258971 | 0.97 | 2395586 | 155.40 | 0.97 | 0.50 |
| F40 | mat | 297578 | 0.97 | 2064557 | 170.90 | 0.98 | 0.52 |
|  | pat | 376158 | 0.97 | 2011286 | 180.45 | 0.96 | 0.48 |

Notes: N50 is an indicator of haplotyping performance, defined as the block length at which the sum of block length of that block and larger blocks represents 50% of the overall phased.
