# Supplementary material for: Noninvasive prenatal diagnosis of monogenic disorders based on direct haplotype phasing through targeted linked-read sequencing
Source: BMC Med Genomics. 2021 Oct 9;14:244. doi: 10.1186/s12920-021-01091-x (PMC8502361; doi:10.1186/s12920-021-01091-x)
Supplement: Supplementary file 2 — Additional file 2: Figure S1. The NIPD results of α-thalassemia and β-thalassemia [file 12920_2021_1091_MOESM2_ESM.docx]

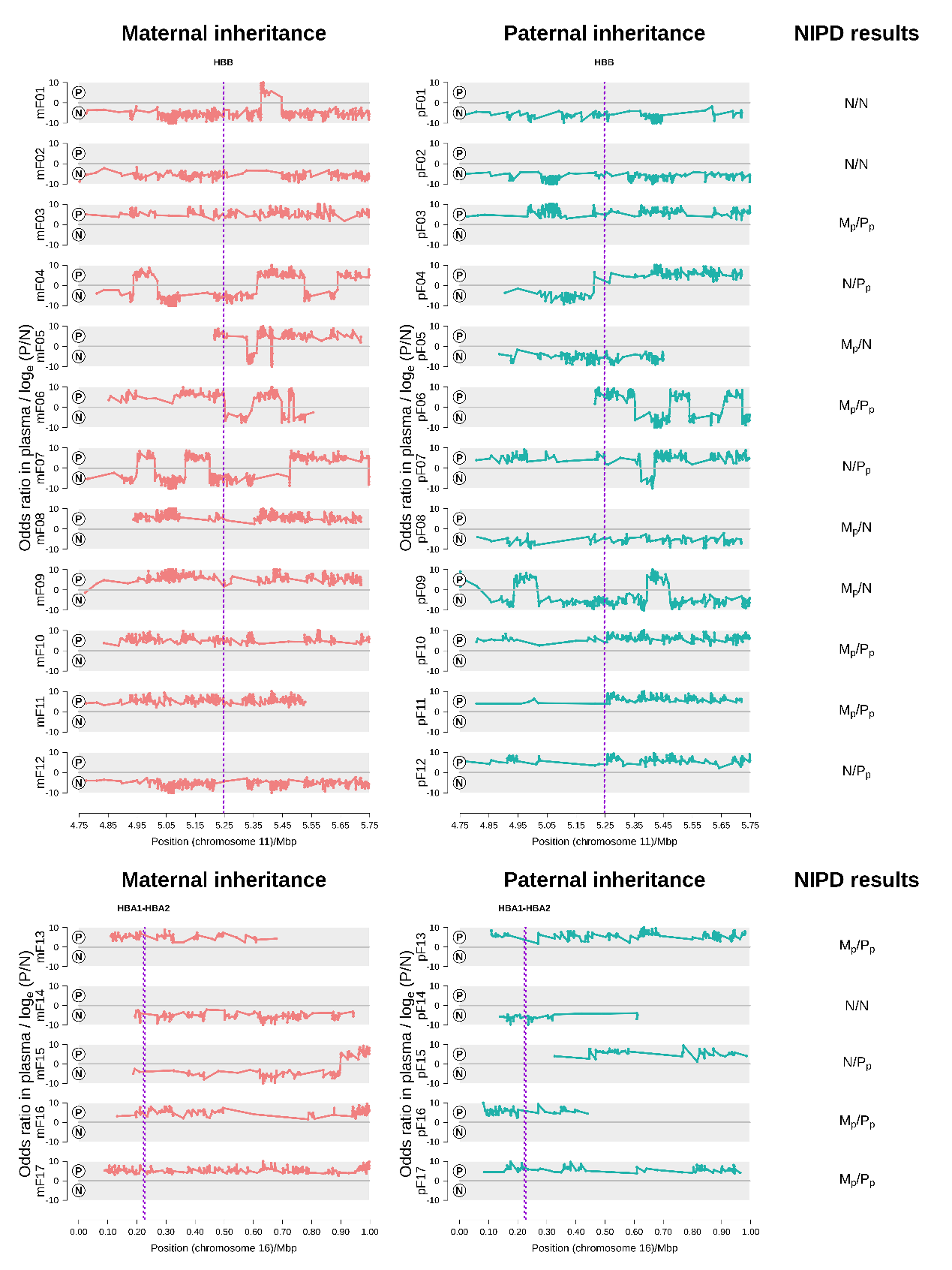


**Additional file 2: Figure S1. The NIPD results of α-thalassemia and β-thalassemia.**

The x-axis indicates the genomic coordinates of the target region (Mbp), the y-axis indicates the loge of the odds ratio of the pathogenicity for each informative SNP, and each red/green line connecting consecutive SNPs (represented as dots) indicates a maternal/paternal haplotype transmission path to the fetus. The purple vertical lines indicate the target genes (*HBB and HBA*).

The paths above zero (gray lines) show fetus inherited pathogenic haplotypes (P) from parents. The paths below zero (gray lines) exhibit fetus inherited normal haplotype (N) from parents. M_p_, maternal pathogenic haplotype, P_p_, paternal pathogenic haplotype; N, normal haplotype.

For mF06, the fetal haplotype block (chr11: 4,852,009 -5,250,551) inferred by NIPD spans the whole *HBB* gene (chr11:5,246,694-5,248,301). The fetal genotype at the pathogenic site is the state of the haplotype block (pathogenic). Therefore, we conclude that the fetus inherited the maternal pathogenic haplotype.

For pF15, the pathogenic variant can be covered by the paternal haplotype phased by linked-reads sequencing, but cannot be covered by the fetal haplotype block inferred by NIPD. In our algorithm (PMID: 29317692), we determined paternal inheritance using paternal informative SNPs, which are heterozygous in the father but homozygous in the mother. For pF15, there was no paternal informative SNPs upstream of the pathogenic variant. This problem can be resolved by increasing the number of informative SNPs flanking the target gene through expansion of the target region. We also queried the deCODE database (PMID: 20981099) for data on the recombination activity at the at the *HBA* loci. However, the deCODE database does not contain data for the recombination rate across the region 500 kb up- and downstream of the *HBA* locus. Therefore, for pF15, we can still safely assume that the phased fetal haplotype block is linked to the adjacent pathogenic site. The fetal genotype at the pathogenic site is the state of the fetal haplotype block (pathogenic).
