# Supplementary material for: Noninvasive prenatal diagnosis of monogenic disorders based on direct haplotype phasing through targeted linked-read sequencing
Source: BMC Med Genomics. 2021 Oct 9;14:244. doi: 10.1186/s12920-021-01091-x (PMC8502361; doi:10.1186/s12920-021-01091-x)
Supplement: Supplementary file 4 — Additional file 4: Figure S3. The NIPD results of PKU, ARPKD and DFNB1A [file 12920_2021_1091_MOESM4_ESM.docx]

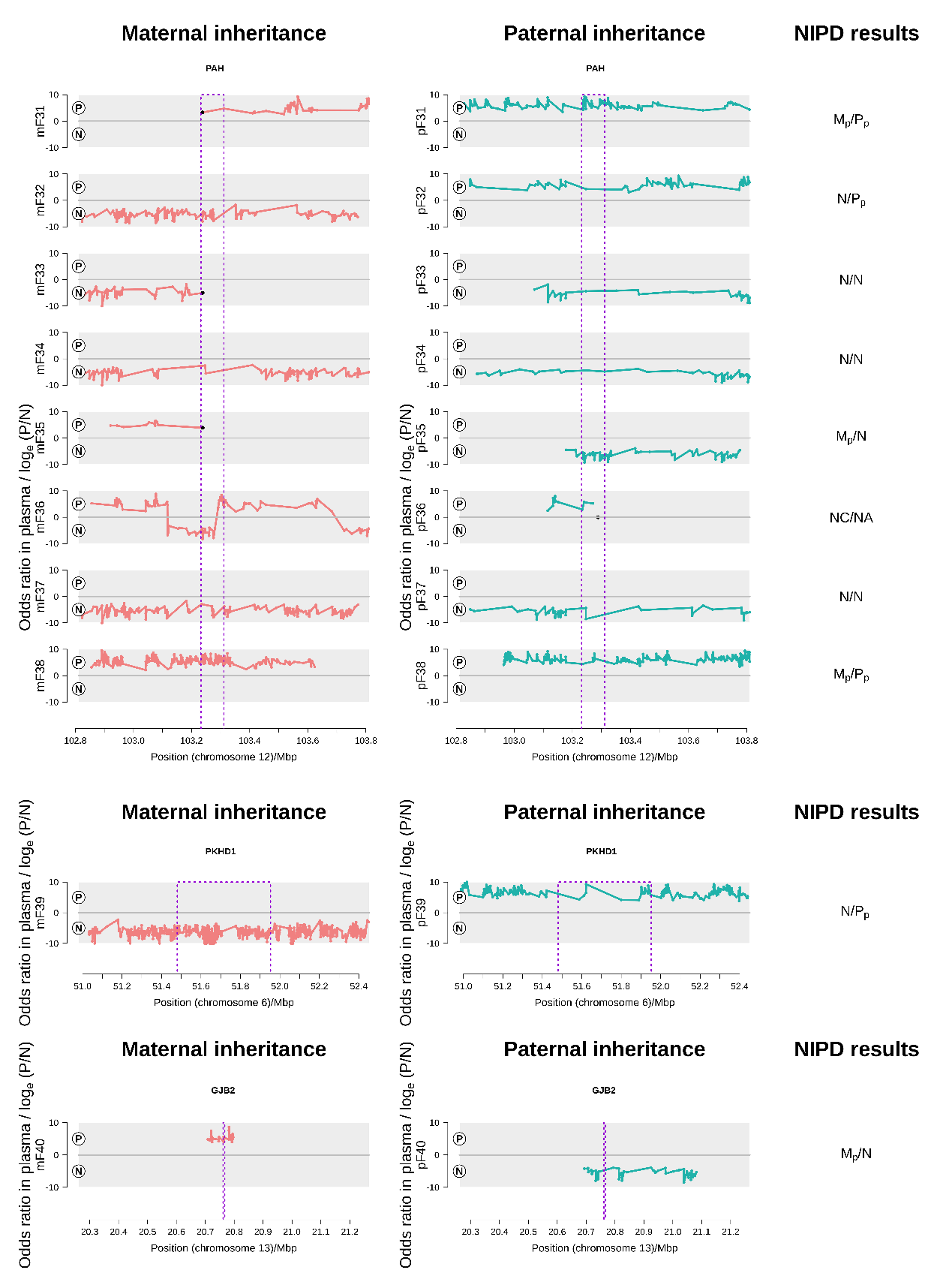


**Additional file 4: Figure S3. The NIPD results of PKU, ARPKD and DFNB1A.**

The x-axis indicates the genomic coordinates of the target region (Mbp), the y-axis indicates the loge of the odds ratio of the pathogenicity for each informative SNP, and each red/green line connecting consecutive SNPs (represented as dots) indicates a maternal/paternal haplotype transmission path to the fetus. The purple vertical lines indicate the target genes (*PAH，PKHD1 and GJB2*). The paths above zero (gray lines) show fetus inherited pathogenic haplotypes (P) from parents. The paths below zero (gray lines) exhibit fetus inherited normal haplotype (N) from parents. NC represents no-call. NA, not applicable, M_p_, maternal pathogenic haplotype, P_p_, paternal pathogenic haplotype; N, normal haplotype.
